# Reactivity of Atomically Functionalized C-Doped Boron Nitride Nanoribbons and Their Interaction with Organosulfur Compounds

**DOI:** 10.3390/nano9030452

**Published:** 2019-03-18

**Authors:** Francisco Villanueva-Mejia, Pedro Navarro-Santos, Peter Ludwig Rodríguez-Kessler, Rafael Herrera-Bucio, José Luis Rivera

**Affiliations:** 1 Instituto de Investigaciones Químico Biológicas, Universidad Michoacana de San Nicolás de Hidalgo, Francisco J. Múgica, s/n, Morelia 58030, Michoacán, Mexico; fvillamejia@gmail.com (F.V.-M.); rhbucio@umich.mx (R.H.-B.); 2 Laboratorio de Cómputo de Alto Desempeño, CONACYT-Universidad Michoacana de San Nicolás de Hidalgo, Edif. B-1, Ciudad Universitaria, Francisco J. Múgica, s/n, Morelia 58030, Michoacán, Mexico; 3 Departamento de Física Aplicada, Centro de Investigación y de Estudios Avanzados, Unidad Mérida, Km 6 Antigua Carretera Progreso, Apdo. Postal 73, Cordemex, Mérida 97310, Yucatán, Mexico; peter.rodriguez@cinvestav.mx; 4 Facultad de Ingeniería Química, Universidad Michoacana de San Nicolás de Hidalgo, Francisco J. Múgica, s/n, Morelia 58030, Michoacán, Mexico

**Keywords:** boron nitride nanoribbons, Fukui functions, thiophene, DBT, 4,6-DMDBT

## Abstract

The electronic and reactivity properties of carbon doped (C-doped) boron nitride nanoribbons (BNNRs) as a function of the carbon concentration were investigated in the framework of the density functional theory within the generalized gradient approximation. We found that the main routes to stabilize energetically the C-doped BNNRs involve substituting boron atoms near the edges. However, the effect of doping on the electronic properties depends of the sublattice where the C atoms are located; for instance, negative doping (partial occupations of electronic states) is found replacing B atoms, whereas positive doping (partial inoccupation of electronic states) is found when replacing N atoms with respect to the pristine BNNRs. Independently of the even or odd number of dopants of the C-doped BNNRs studied in this work, the solutions of the Kohn Sham equations suggest that the most stable solution is the magnetic one. The reactivity of the C-doped BNNRs is inferred from results of the dual descriptor, and it turns out that the main electrophilic sites are located near the dopants along the C-doped BNNRs. The reactivity of these nanostructures is tested by calculating the interaction energy between undesirable organosulfur compounds present in oil fuels on the C-doped BNNRs, finding that organosulfur compounds prefer to interact over nanosurfaces with dopants substituted on the B sublattice of the C-doped BNNRs. Most importantly, the selective C doping on the BNNRs offers the opportunity to tune the properties of the BNNRs to fit novel technological applications.

## 1. Introduction

Over the last decade, part of the scientific community has focused on the study of matter with low dimensionality, demonstrating the importance of atomically thin, long and narrow structures. The discovery of nanomaterials with novel properties is resulting in intense research labor toward the understanding of these materials, which could be used in promising novel applications. An effort to understand their behavior is being made through the application of computational simulations, which are a value tool to predict their physicochemical properties and, moreover, providing testing routes to generate such structures where smaller components imply greater experimental challenges. Among such novel structures, carbon-based materials are promising at nanoscale level, and exhibit remarkable features. With regard to their tunable electronic properties, which depend in part on their chirality [1,2], carbon nanotubes (CNTs) with armchair chirality (*n*,*n*) are metallic, while zigzag CNTs (*n*,0) can be metallic or semiconducting [3]; two-dimensional carbon-based nanostructures, such as graphene and carbon nanoribbons, are semiconducting [4,5].

Other nanostructure analogues to graphene have emerged that show promising tunable electronic properties; these include boron nitride (BN)-based materials. BN is more likely to be found in a hexagonal form [6] (*h*-BN) that resembles the honeycomb graphene, although it can also be found in sphalerite-type (*β*-BN) forms [7] related to cubic diamond, and wurtzite-type *γ*-BN forms [8] related to hexagonal diamond. Similar to graphite, *h*-BN presents strong bonding within the atomic layers and weak interactions between layers, resulting in similar interlayer distances between the sheets [9,10]. One difference of *h*-BN with respect to graphite is that the planar-fused six-membered rings are stacked directly on top of each other, with boron atoms in one layer serving as the nearest neighbors to the nitrogen atoms in the adjacent layers, with the interlayer interactions between the B and N of Lewis acid being of base type [11]; meanwhile, in the graphitic form of carbon, there is an offset in the adjacent layers of carbon atoms [4]. Concerning its electronic properties, *h*-BN is a great isolator, while graphene is semiconducting [12,13]. In addition, BN is found in other allotropic forms, such as BN nanotubes [14]. Moreover, recent calculations have predicted the exciting potential of BN-based materials in spintronics [15,16,17], and one layered boron nitride sheet a few nanometers in width, in particular—namely, boron nitride nanoribbons (BNNRs)—because of its magnetic and electrical properties. These are mainly found as BNNRs with zigzag conformations [17]. Since BNNRs were first synthetized [18], they have opened a range of possibilities for technological applications [19], e.g., as selective adsorbent materials.

The remaining undesirable molecules present in oil fuels, such as gasoline, highway diesel and jet fuel include the organosulfur compounds [20]. A direct environmental impact occurs when lower sulfur levels are achieved, due to the increasing operational costs of traditional hydrodesulfurization processes [21,22]. In this way, alternative techniques, e.g., by using adsorbent materials [23,24,25,26], have been tested to selectively eliminate residual organosulfur compounds. Therefore, carbon doped (C-doped) BNNRs may be an attractive adsorbent material because of their tunable electronic properties. To identify potentially promising adsorbent materials for eco-friendly applications, reliable information about their electronic structure is mandatory.

In this work, the electronic properties and reactivity of C-doped BNNRs when varying the carbon concentration were studied in the framework of the density functional theory. Our research work is structured as follows: The employed methodology is described in Section 2; Section 3 includes the main results of our work, starting with an energetic and stability analysis, and the electronic properties and reactivity of the considered C-doped BNNRs; the last part of Section 3 shows the results of the interaction of organosulfur compounds on the most reactive sites of the C-doped BNNRs. Finally, in Section 4, a summary of our main findings and conclusions are presented.

## 2. Computational Details

**Figure 1:**
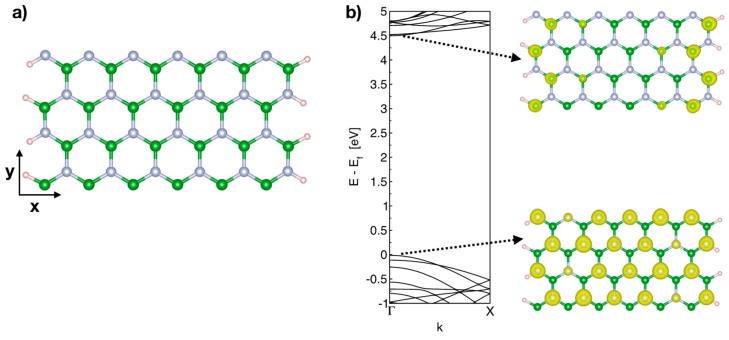
(**a**) Schematic of 12 × 2 BNNR passivated by hydrogen atoms, the periodic direction is along the *y* axis. (**b**) Band structure of the 12 × 2 pristine BNNR and the wave function integrated for the lowest unoccupied band (right upper) and the highest occupied state (right lower). Green, light blue and pink spheres represent boron, nitrogen and hydrogen atoms, respectively.

All calculations for this work were performed by using the Vienna ab initio Simulation Package (VASP) (ABACUS, CINVESTAV, Estado de Mexico, Mexico) [27,28]. The geometry optimizations of all systems were relaxed at DFT level without imposing any symmetry constraint, by using the generalized gradient approximation in the form proposed by Perdew, Burke and Ernzerhof [29]. The interaction between ionic cores and valence electrons is described by the projector-augmented wave method, as implemented in the VASP code [30,31]. The energy cutoff for the wave-plane basis set was set to 450 eV, and the conjugate gradient method was used until the forces on each atom were lower than 0.01 eV/Å. The dimensions of the orthorhombic unit cell in the non-periodic axis were large enough to avoid interactions between images and edged hydrogen atoms. Herein, a space of ~10 Å along the non-periodic directions was fixed, while through the periodic dimension (along y-axis of Figure 1a), the unit cells were optimized until the cell total stress was close to a minimum. According to cell dimensions, a few *k*-points are needed to sample the Brillouin zone in reciprocal space. Thus, a grid of 1 × 20 × 1 points, the Monkhorst-Pack, and a plane-wave cutoff energy of 500 eV represents a good compromise between precision and computing time for the optimization of the electronic density at each relaxation step. We searched for spin-polarized and non-spin-polarized solutions of the Kohn–Sham equations for the bare and C-doped nanostructures of BNNRs, finding significant differences in the C-doped BNNRs in agreement with the literature [32], such calculations were performed spin polarized, with the total energy per unit cell being more negative than the non-polarized approximation, so that the components of the spin density were distinct from zero in the cases of the magnetic nanoribbons. In spin restricted or unrestricted calculations, closed shell systems are characterized by having doubly occupied molecular orbitals (restricted case), whereas in open shell systems, the spin degree of freedom makes it possible to obtain a complete set of orbitals for *α*-spin electrons and another set for *β*-spin electrons. In this way, our results, presented in the next section, will be discussed in terms of the spin-polarized solution. The present calculation details were also used to describe systems that can be periodic in one dimension, giving reasonable results in good agreement with the experimental data [33,34,35].

To explore the reactivity of the C-doped BNNRs, we used two appropriate reactivity descriptors to identify the most active sites along the BNNRs, covering both covalent and non-covalent interactions. The first is the electrostatic potential, which is defined as

(1) $$V\left(r\right)=\underset{A}{\sum }\frac{Z_{A}R_{A}}{\left|R_{A}-r\right|}-\int^{\text{​}}\frac{\rho \left(r^{\prime }\right)}{\left|r^{\prime }-r\right|}dr^{\prime }$$

which provided us the response of the electron density when a positive unit charge is approaching, plotted in a color scheme. The second one is the Fukui or frontier functions, which are defined in a finite difference approximation as follows:

(2) $$f_{v,N}^{+}\left(r\right)=\rho_{v,N+1}\left(r\right)-\rho_{v,N}\left(r\right)$$

(3) $$f_{v,N}^{-}\left(r\right)=\rho_{v,N}\left(r\right)-\rho_{v,N-1}\left(r\right)$$

(4) $$f_{v,N}^{o}\left(r\right)=\frac{1}{2}\left[\rho_{v,N+1}\left(r\right)-\rho_{v,N-1}\left(r\right)\right]$$

where $\rho_{v,\;N+1}\left(r\right)$, $\rho_{v,\;N}\left(r\right)$ and $\rho_{v,\;N-1}\left(r\right)$ are the electronic densities of a system with *N* + 1, *N*, and *N* − 1 electrons, respectively, all at the ground state geometry of the *N* electron system. Referring to the Maxwell relation, f(r) is related to the change of density in response to changes in the number of electrons *N* [36]. Expressions (2)–(4) may be respectively evaluated for: nucleophilic attack representing the response of the BNNRs to incoming charge, electrophilic attack that means sites of the BNNRs may lose charge; and for free radical attacks [37]. In the frozen orbital approximation, these quantities can be approximated with the square of the lowest unoccupied (LUMO) and highest occupied molecular orbitals (HOMO) [36]:

(5) $$f^{+}\left(r\right)={\left|\phi_{v,N}^{LUMO}\left(r\right)\right|}^{2}=\rho_{v,N}^{LUMO}\left(r\right)$$

(6) $$f^{-}\left(r\right)={\left|\phi_{v,N}^{HOMO}\left(r\right)\right|}^{2}=\rho_{v,N}^{HOMO}\left(r\right)$$

To approximate these quantities, we considered the electron density of the lowest unoccupied electronic band above the Fermi level (E_F_) and the highest occupied electronic band below E_F_, respectively, in order to compute the $f^{+}\left(r\right)$ and $f^{-}\left(r\right)$ flavors of the Fukui functions to covalently analyze the reactivity of the C-doped BNNRs.

The molecular electrostatic potential of the BNNRs is computed in the VASP package, while the Fukui functions are calculated by using the electronic densities applying Equations (5) and (6) visualized using the VESTA package [38]. Once the Fukui functions have been obtained, the dual descriptor is computed.

## 3. Results and Discussion

### 3.1. Energetic Stability and Electronic Properties of C-Doped BNNRs

Firstly, the BNNRs considered in this work were built by passivating the edged N and B atoms with one hydrogen atom. Figure 1a shows the optimized structure of the pristine BNNR of size 12 × 2 (following a convention of size *M* × *N*, with *M* dimer lines along the width of the BNNR and *N* being the number of six-member rings along the periodic direction). Although the BNNRs analyzed in this work go from 0.4 nm (4 × 2) to 3.5 nm (30 × 2), after the optimization routine, the most stable configuration of the pristine BNNR has similar a length parameter of *y* = 8.70 Å (along the periodic direction), meanwhile the optimized unit cell length of the C-doped BNNR is slightly reduced (up to 0.2 Å) due to the doping. In all cases, the optimized geometries show average bond lengths of 1.45, 1.57 and 1.43 Å for the pairs B–N, C–B and C–N, respectively. In the case of the pristine BNNR of length 12 × 2, our results confirm its characteristic constant energy gap found in BNNRs [39]. The electronic properties of the C-doped BNNRs were compared within the local density approximation (LDA) and the gradient generalized approximation (GGA), finding in general that the treatment within the LDA underestimates the band gaps of the C-doped BNNRs, as has previously been reported for pristine BNNRs [39].

From Figure 1b, we can observe that the conduction band minimum (CBM) of the pristine BNNR is composed of edge states localized at boron atoms, while in the highest occupied state, the valence band maximum (VBM) has electronic states localized at nitrogen atoms throughout the ribbon.

Considering the C-doped BNNRs, the effect of three variables were studied on the properties of the BNNRs: the concentrations of dopants (from 0.8 to 6.25%), and the relative position of impurities along the nanoribbon, for instance, near the edges (E) and center (C). Bearing in mind that in the peculiar distribution of the BN lattice shown in Figure 1a, one can observe that the nearest neighbor to each B (N) atom belongs to the same sublattice. Therefore, the third variable to consider in this work depends on what atom C is replaced with, for instance, nitrogen (N) or boron (B). For simplicity, the C-doped are identified as BNNR_XY according to the replaced atom of the pristine lattice (X = N, B) and the relative position of the dopant along the nanoribbons (Y = E, C).

**Figure 2:**
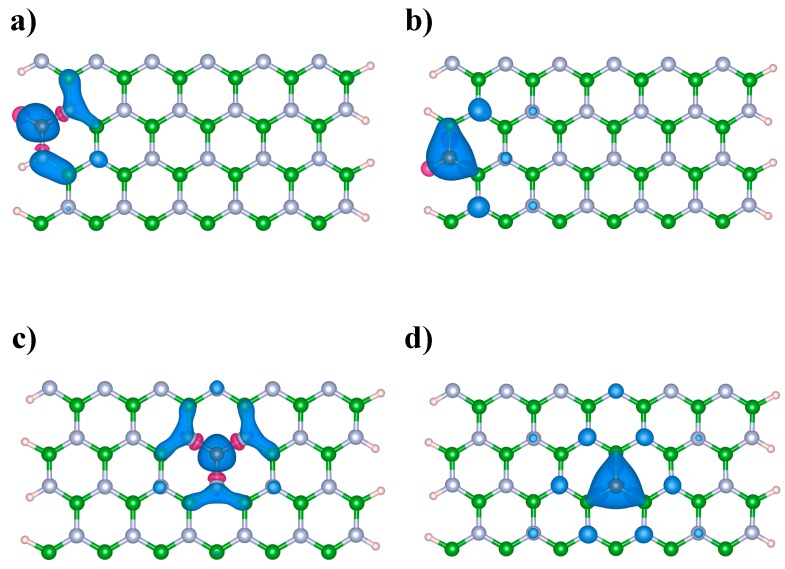
Spin density of the C-doped BNNRs: (**a**) BNNR_BE, (**b**) BNNR_NE, (**c**) BNNR_BC and (**d**) BNNR_NC of size 12 × 2. Light blue, green, brown and pink balls represent N, B, C and H atoms respectively.

We analyzed the spin-polarized and non-spin-polarized solutions of the Kohn–Sham equations, finding significant differences among all the C-doped BNNRs studied in this work. These findings were expected due to the unpaired number of electrons caused for the doping, particularly when odd atoms are replaced from the pristine BNNRs, e.g., Du et al., reported the spontaneous magnetization of BNNRs with a single C-substitution [32]. However, when even numbers of atoms were substituted in the bare BNNRs, magnetic properties were still observed. Figure 2 shows, through the use of a color scheme, the spin density of C-doped BNNRs; we can observe from this figure that, independently of the relative position of the C atom and what the atom was substituted for, the spin density is found near the dopants, even for the cases of C-doped BNNRs with higher C doping (see Supplementary Materials).

Spin polarized DFT has been described as a useful tool for analyzing chemical reactivity [40], because it distinguishes between the changes produced by the charge transfer of interacting species and the changes produced by the distribution of electron density. In fact, molecular systems susceptible to changes in their spin state as result of a chemical reaction have been studied in order to correlate their chemical reactivity and spin-philicity-donacity [41]. Therefore, C-doped BNNRs can drive spin polarization processes (e.g., photophysical changes) linking states of different multiplicity near the dopants.

To analyze the energetic stability of the C-doped BNNRs, Table 1 shows the cohesive energy $\left(E_{C}\right)$ of the pristine and C-doped BNNRs of widths M = 12, 16 and 24. The $E_{C}$ is the energy required to disassemble a molecular system into its constituent atoms, so that a bound (stable) state has a positive value of $E_{C}$, representing the energy gained during the formation of such a bound state. On one hand, the $E_{C}$ of pristine BNNRs increases with respect to the width and length of the ribbon; one can consider the formation of a *h*-BN sheet to be the upper limit. In this way, our results are in good agreement with values of $E_{C}$ calculated for *h*-BN sheets (7.095 eV atom^−1^) and cubic BN (7.126 eV atom^−1^) [9]. On the other hand, it is observed that the carbon doping slightly decreases the cohesive energy compared to the pristine BNNRs; this energy decrement has been reported in other doped nanoribbons [42], and this reduction becomes less important when the width of the system increases. Particularly, the energy decreases as BNNR_NE > BNNR_BE > BNNR_NC > BNNR_BC, indicating that substitutions on the edges and replacing N atoms are key to the stability routes of C-doped BNNRs. However, $E_{C}$ computations for the “average” atom bear little significance for structures of different composition. Therefore, we adopt the approach customarily used in ternary phase thermodynamics to account for chemical composition and to analyze the relative stability of endohedral silicon nanowires [43] and other 1D nanoribbons [35,42,44]. Table 1 also shows the molar Gibbs free energy (δG) listed in parenthesis, the δG is given by

(7) $$\delta G=E\left(x\right)+\overset{n}{\underset{i=1}{\sum }}X_{i}\mu_{i}$$

where E(x) is the binding energy per atom of the C-doped BNNRs. $x_{i}$ corresponds to the molar fraction of the conformant components (H, N, B, C), which satisfies $\sum^{\text{​}}X_{i}=1$. The chemical potential ($x_{i}$) can be approximated as the binding energy per atom of the singlet ground state of the H_2_ and N_2_, the triplet ground state of the B_2_ molecule, and the cohesive energy per atom of the graphene sheet, respectively. It should be mentioned that this treatment gives a qualitative measure of the relative stability, while neglecting thermal and substrate effects and zero-point energy corrections, as has previously been successfully presented [45]. Here, it is observed that the results of δG confirm that doping near the edges is a more energetically stable route to stabilize the C-doped BNNRs than substitutions near their center. However, considering the composition of the C-doped BNNRs, the BNNR_BE are up to 25 meV/atom more stable that the BNNR_NE ones. However, increasing the doping concentration, the most energetically (thermodynamically) stable C-doped BNNRs are found by substituting 4 N atoms for C, being up to 93 (37) meV/atom more stable than the structures of 4 B atoms substituted for C.

**Figure 3:**
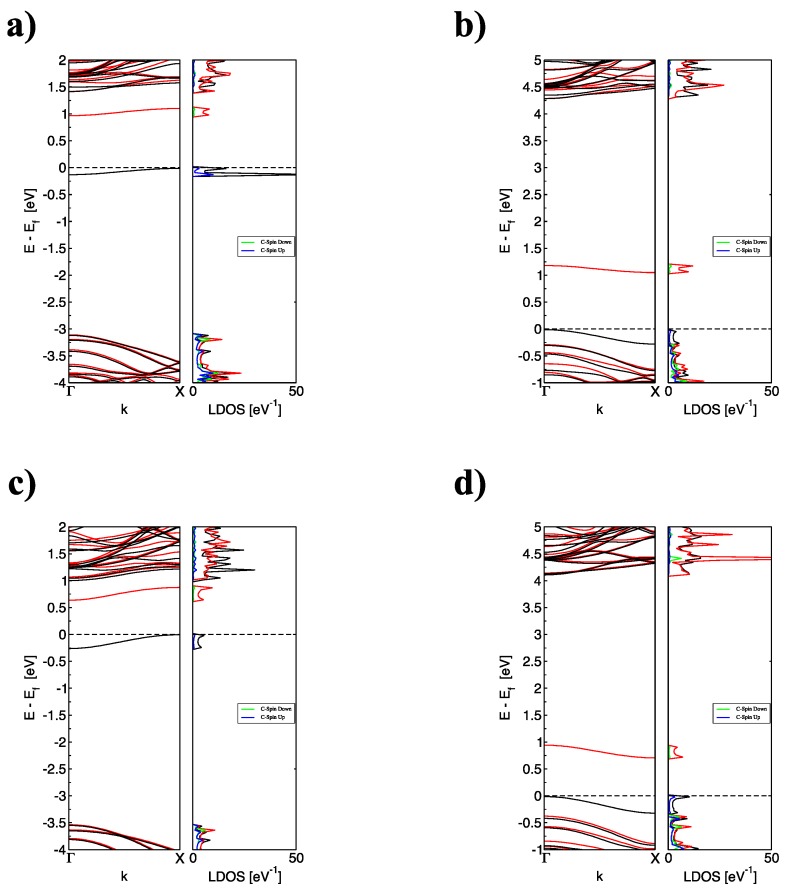
(color online) The corresponding band structure and total density of states of (**a**) BNNR_BE, (**b**) BNNR_NE, (**c**) BNNR_BC and (**d**) BNNR_NC of size 12 × 2 with one dopant; black lines indicate the contribution of Spin Up and red lines indicate the contribution of Spin Down; the Fermi level is indicated as the black dotted line.

The electronic structure of the C-doped BNNRs is inferred from the band structure and the total density of states. These quantities were calculated by integration over the Brillouin zone using a Monkhorst–Pack grid two times denser than that employed for the energy calculations, improving the convergence of the integrals using a gaussian smearing of the energy levels with a width of 0.01 eV. Figure 3 presents the band structure and total density of states of C-doped BNNRs of size 12 × 2. 

Clearly, the substitution of B causes a significant upshift (up to 3.5 eV for the BNNR_BC) with respect to the pristine BNNR, whereas the Fermi level $\left(E_{F}\right)$ is lowered (~1 eV) when N atoms are substituted, independently of the relative position of N on the BNNR. These findings are in agreement with previous calculations [32]. The partially occupied (unoccupied) band may be explained by the addition (subtraction) of one electron when a B(N) is replaced in the pristine BNNR; the flatness of the occupied bands and the localized density of the states below $E_{F}$ indicates that the corresponding electron state is strongly localized at the substitution site, in accordance with Figure 1b for the pristine case, being only one electronic band moving above $E_{F}$ (for substitutions on the N sublattice) and below $E_{F}$ (for substitutions on the B sublattice), respectively. 

**Figure 4:**
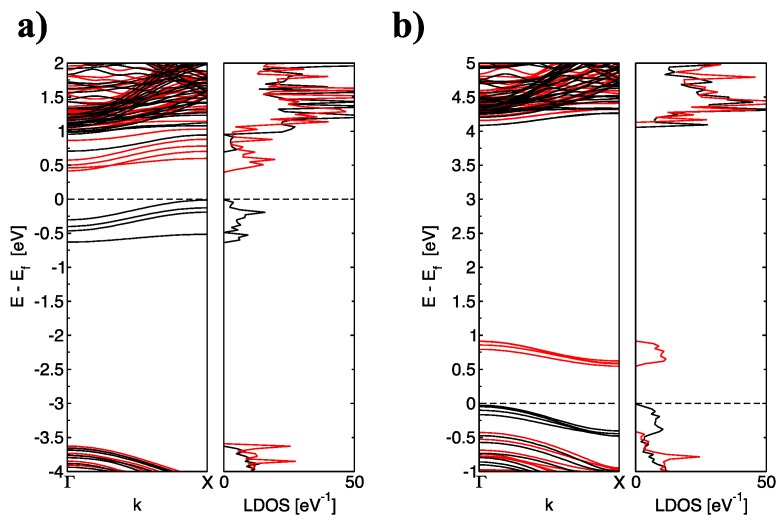
(color online) The corresponding band structure and total density of states of (**a**) BNNR_4B and (**b**) BNNR_4N of size 30 × 2 with 4 dopants substituted in the same sublattice; black lines indicate the contribution of Spin Up and red line indicates the contribution of Spin Down; the Fermi level is indicated as a black dotted line.

An important question is: how are the electronic properties of the C-doped BNNRs modified at higher doping concentrations? To answer this question, Figure 4 shows the electronic properties of the C-doped BNNRs of size 30 × 2 at the highest dopant concentration studied in this work. Interestingly, the spin density remains on alternating even-odd doping numbers, i.e., the magnetic solution of the Kohn Sham equation is 494 meV more stable than the non-magnetic solution when 4 boron atoms are replaced by carbon, and 449 meV more stable when 4 nitrogen atoms are substituted with carbon.

From Figure 4, one can observe the same electron spin in the occupation (inoccupation) of electronic bands for even numbers of dopants substituted on the same sublattice if and only if all C atoms are substituted on the same B(N) sublattice. In fact, one may assume that one occupied (unoccupied) band corresponds to one dopant atom. This can be explained by the even number of electrons added (subtracted) from the pristine lattice of the BNNRs when B(N) atoms are replaced by C atoms. In addition, the upshift of the $E_{F}$ is shown in Figure 4a. In addition, from Figure 4b, the decrease of the $E_{F}$ described previously for narrower C-doped BNNRs can be observed.

We explored other doping distributions along the BNNRs, i.e., random dopant distributions replacing boron and nitrogen atoms at the same time; although very interesting results were obtained, it was difficult to explain the trend in terms of the position and the substituted atom belonging the B or N lattice, which is the aim of this work. However, it is worth mentioning that magnetic properties were observed in such cases.

### 3.2. Reactivity of C-Doped BNNRs

**Figure 5:**
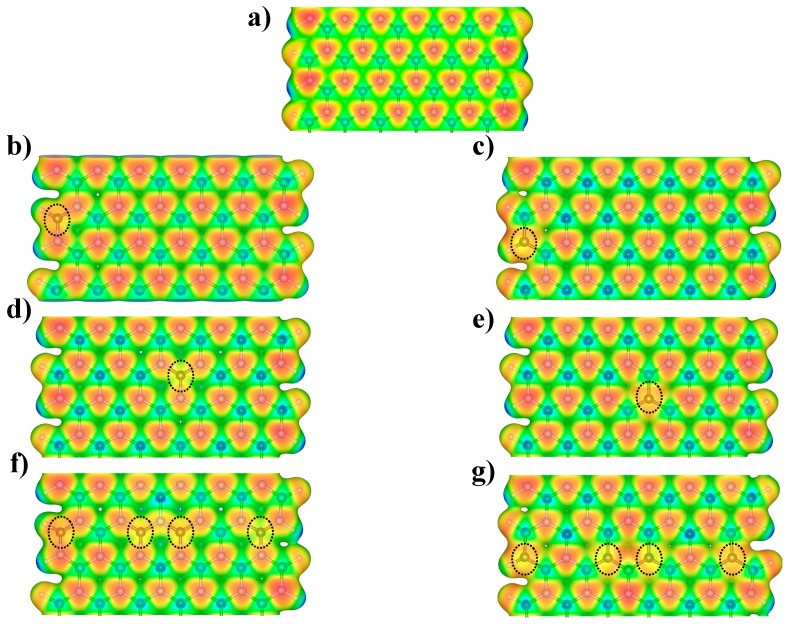
The molecular electrostatic potential on the *ρ*(*r*) = 0.02 a.u. for the (**a**) pristine BNNR, (**b**) BNNR_BE, (**c**) BNNR_NE, (**d**) BNNR_BC, (**e**) BNNR_NC, (**f**) BNNR_4B and (**g**) BNNR_4N respectively. Carbon atoms are highlighted with dotted lines to facilitate their positioning along the C-doped BNNRs.

To understand how the introduction of charge defects via carbon doping modifies the chemical reactivity of the BNNRs, the computed MEP of the BNNR_BE, BNNR_NE, BNNR_BC and BNNR_NC, as well as higher doping concentration, are mapped in Figure 5. Blue regions represent zones in which a positive charge is expected, whereas the most negative values, which represent zones that a proton can interact with, are colored in red. The MEP of the pristine BNNR is shown in Figure 5a; we observed clearly a negative region on the N atoms along the entire nanostructure, increasing slightly at the nitrogen edges. On the other hand, from Figure 5b,d,f, the C-doping on the B lattice gives rise to extended negative regions near the dopants; this fact may be explained because π electrons of the C atom serve as a bridge to delocalizing the electrons of the N atoms. Note that the N atoms have a higher tendency to attract electrons than C, so that the MEP near the carbon atoms is less negative than their neighboring nitrogen atoms.

On the other hand, we can observe from Figure 5c,e,g that C-doping on the N lattice gives rise to localized negative regions around its neighboring B atoms. In these cases, the C atoms have less negative potential than N. This fact may be explained by the fact that π electrons of the C tend to interact with the unoccupied orbital of the neighboring B atoms. From these results, one can observe that C doping redistributes the electron density of the bare BNNRs, promoting negative regions along the BNNRs.

Once the Fukui functions are obtained, the Dual Descriptor [46] (DD) of the C-doped BNNRs was computed as the difference between $f^{+}\left(r\right)$ and $f^{-}\left(r\right)$. The DD is a reactivity index has been successfully applied to predict the most reactive sites of BN nanotubes [47], fullerenes [48], heterogeneous catalytic systems [49], among other nanostructures. The DD is a local chemical reactivity index that can be applied to simultaneously obtain the nucleophilic (*f*^2^(*r*) > 0) and electrophilic (*f*^2^(*r*) < 0) behavior of a molecular system at point r. When *f*^2^(*r*) > 0, the process is driven by a nucleophilic attack on the atom *i*; then, the atom *i* acts as an electrophilic species.

**Figure 6:**
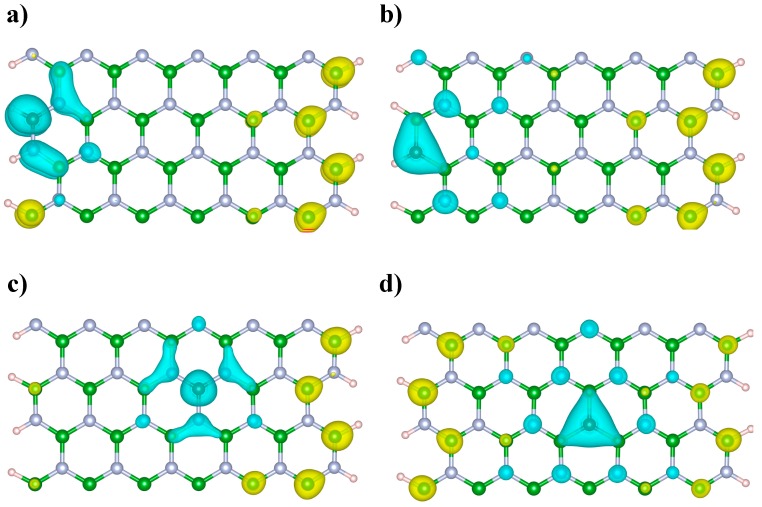
Dual descriptor of the C-doped BNNRs (**a**) BNNR_BE, (**b**) BNNR_NE, (**c**) BNNR_BC and (**d**) BNNR_NC. Green, light blue, brown and pink spheres represent boron, nitrogen, carbon and hydrogen atoms respectively. The light blue lobes represent regions for nucleophilic attacks (*f*^2^(*r*) > 0) and the yellow lobes represent regions for electrophilic attacks (*f*^2^(*r*) < 0).

It is possible to qualitatively compare the most reactive sites of the C-doped BNNRs from Figure 6. We can observe from Figure 6 that the most nucleophilic lobes are located near the dopant independently of its location along the BNNRs. Note from Figure 6b,d that, at C substitutions on the N lattice, the nearest boron atoms are involved in the most nucleophilic lobes, whereas, from Figure 6a,c it can be observed that the most nucleophilic lobes involving the nearest B–N pairs are found around the C atoms. On the other hand, electrophilic attacks are expected on the edged B atoms opposite the dopant location. In general, similar reactive sites were found at higher dopant concentrations (see Supplementary Materials). 

From the reactivity analysis of the C-doped BNNRs, it is important to mention that the DD will help to predict electron-transfer processes occurring due to soft reagents, whereas the MEP is more suitable as an electrostatic property involving charge processes because of hard reagents [50]. In the next section, the reactivity of the C-doped BNNRs will be tested by studying the interaction of thiophene, benzothiophene, dibenzothiophene, 4-methyldibenzothiophene and 4,6-dimethyldibenzothiophene, which are organosulfur compounds present in crude oil and important refining products.

### 3.3. Interaction of Organosulfur Compounds on C-Doped BNNRs

In this section, the interaction energies and the optimized geometries of the complexes formed between thiophene (Thio), benzothiophene (B-Thio), and the refractory molecules dibenzothiophene (DBT), 4-methyldibenzothiophene (4-MDBT) and 4,6-dimethyldibenzothiophene (4,6-DMDBT) on the C-doped BNNRs are analyzed to give insights into the capacity of these nanomaterials for possible application in selective adsorption processes, particularly when involving such adsorbed states of the sulfur atom of organosulfur compounds. These organosulfur compounds are the most difficult to remove from oil fuel [21]. Zhang et al. [51] reported an oxidative adsorptive desulfurization process using graphene oxide in the presence of air oxygen under mild conditions; from that report, the removal efficiency of the sulfur compounds on graphene oxide followed the order: Thio > DBT > 4,6-DMDBT > B-Thio.

Based on previous reports, we know that the most reactive sites of the DBT are the sulfur atom (which may behave as a nucleophile) and its aromatic rings, which can also donate charge, and eventually their hydrogen atoms could accept charge [52,53,54]. Despite these facts, we built complexes between the organosulfur compounds and the C-doped BNNRs, considering several adsorption modes of the organosulfur compounds to explore their affinity on all the selected potentially active sites, i.e., when adsorption takes place through the sulfur atom perpendicularly oriented to the surface (namely, <<1>>). In the competitive case, the hydrogens of the aromatic rings of organosulfur molecules are oriented to the surface (namely, <<2>>) in consideration of the initial parallel position of the organosulfur compounds with respect to the C-doped BNNRs. Although none of the parallel conformations converged to energetically stable states by using the PBE function [29], in contrast, all of them are stable considering the semi-empirical DFT-D2 long-range dispersion correction [55], included in the following results. The interaction energy $\left(E_{i}\right)$ is computed by using the following equation:

(8) $$E_{i}=E_{complex}-\left(E_{BNNR_{XY}}+E_{org\_mol}\right)$$

where $E_{complex}$ is the energy of the optimized complex formed between the organosulfur molecule and the C-doped BNNR, $E_{BNNR_{XY}}$ is the energy of the optimized C-doped BNNR, and $E_{org\_mol}$ is the energy of the optimized organosulfur molecule in the gas phase.

**Figure 7:**
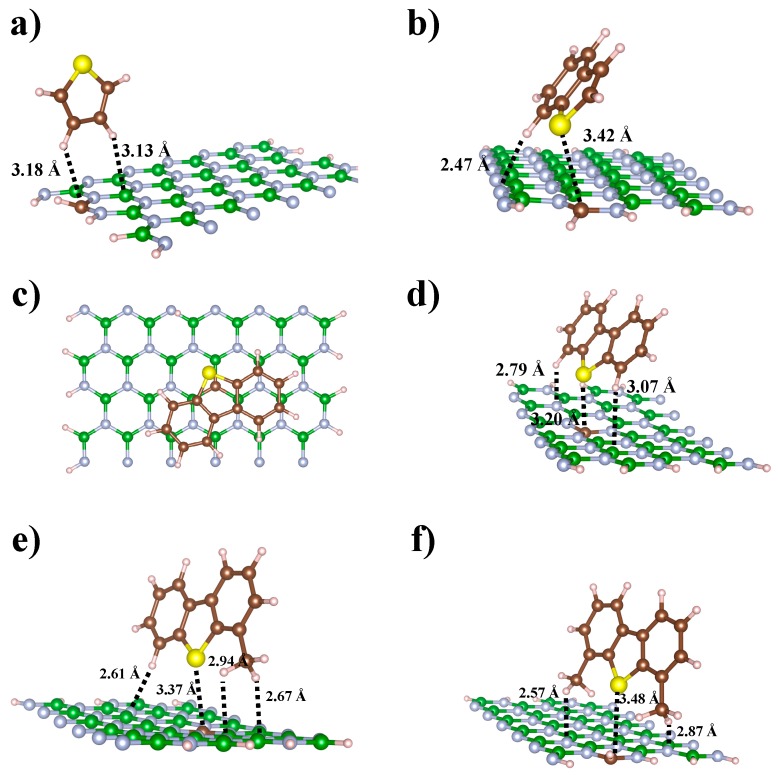
Complexes formed between the organosulfur compounds: Tio, B-Tio, DBT, 4-MDBT and 4,6-DMDBT on the C-doped BNNRs: (**a**) BNNR_BE, (**b**) BNNR_BE, (**c**) BNNR_NC and (**d**) BNNR_BC, (**e**) BNNR_BC and (**f**) BNNR_BE. Light blue, green, brown, yellow and pink balls represent N, B, C, S, and H atoms respectively.

Table 2 collects the interaction energy values of the most stable configurations concerning the adsorbed states formed between organosulfur molecules and C-doped BNNRs. As can be observed from Table 2, for the complexes between the Thio and the C-doped BNNRs, the $E_{i}$ is slightly more stable (up to 1.8 kcal/mol) in the <<2>> configuration than those in the <<1>> complexation states. In particular, the adsorbed state of the lowest $E_{i}$ involves the hydrogens of Thio, perpendicularly oriented to the BNNR_BE. From Figure 7a, it can be observed that tight interactions in such adsorbed states involve the dopant and its neighbor B atom of the surface, the interaction lengths are 3.18 and 3.13 Å, respectively.

On the other hand, for complexes of organosulfur compounds containing benzene rings, it was found that B-Thio and DBT adsorption geometries are quite similar on the active sites of the C-doped BNNR with impurities substituted on the boron lattice, although the adsorption energy of the DBT molecule is slightly higher. The magnitude of $E_{i}$ in conformation <<1>> for B-Thio and DBT is higher than the complexes of <<2>>, independently of the arrangement of dopants in the BNNRs. From the adsorbed state of the highest $E_{i}$ for B-Thio, it was found that the B-Thio molecule was tilted (~51° with respect to the surface plane), tight interactions were found in that adsorbed state due to the formation of a hydrogen bond (2.47 Å) between the H of the aromatic ring with a N atom of the BNNR_BC and the direct interaction of the lone pairs of the sulfur atom with the most electrophilic region of the BNNR_BC. 

Based on the conformations of the adsorbed state of the DBT molecule, it was found that DBT is adsorbed in both the planes parallel and perpendicular to the C-doped BNNRs; the adsorption geometries are shown in Figure 7c,d, respectively. In the parallel plane adsorbed geometry, the DBT is stabilized due to the fact that sulfur pairs participate less in the electrophilic zones of the surface, with the long-range interaction between the π-π* system of the thiophene ring of the DBT molecule and the most electrophilic zone of the BNNR_NC being the most important interactions. Indeed, regarding the complexes formed between B-Thio and DBT on the C-doped BNNRs with dopants substituted on the N lattice, they converged to parallel configurations with respect to the surface (see Supplementary Materials for details), involving the direct interaction between the thiophene ring of the organosulfur compound with the most electrophilic region of the BNNRs. This fact is probably due to both of the nearest B atoms to C having one unoccupied π orbital, and then the lack of π electrons of the B – C – B atoms interacts strongly with the aromatic thiophene ring, which is in agreement with results of the MEP isosurfaces shown in Figure 5. Figure 7d shows the complex formed for the DBT on the BNNR_BC; note that the configurations of both aromatic organosulfur molecules considered in this work are tilted in such adsorbed states (~54°), which is in agreement with other complexes involving organosulfur compounds adsorbed on C-based doped nanomaterials [53].

The magnitudes of the $E_{i}$ calculated for 4-MDBT and 4,6-DMDBT are quite similar for the complexes with conformations <<1>> and <<2>>, respectively. We found that 4-MDBT and 4,6-DMDBT interact preferably with BNNRs with dopants on the B lattice than those BNNRs with dopants on the N lattice. Figure 7e shows that hydrogens of the methyl group 4-MDBT are involved in such an adsorbed state, forming C-H^…^N hydrogen bonds of lengths 2.61, 2.64 and 2.94 Å with N atoms of the nanosurface. On the other hand, we know from previous calculations [52] that the intrinsic reactivity of the S atom of 4,6-DMDBT is sterically hindered because of its methyl groups. From Figure 7f, it can be observed that the S atom of the 4,6-DMDBT molecule is more separated than in other complexes from the electrophilic region of the C-doped BNNR. However, strong hydrogen bonds between one hydrogen of both methyl groups and the N atoms of the BNNR_BE are calculated at 2.57 and 2.87 Å, respectively.

Our results for $E_{i}$. calculated in this work are within the conventionally accepted threshold by separating physisorption and chemisorption (EA ~ 0.04–1.75 eV). Qualitatively, from an energetic point of view, the upper limit for a physisorption process is usually rather weak (~0.1 eV) [56], with our results being in quantitative agreement with previous theoretical calculations for the physisorption of Thio and DBT on CNTs, graphene and B-doped CNRs, respectively [56,57,58].

## 4. Conclusions

In this work, we have presented a first principles study of the electronic and reactivity properties of high and low C-doped BNNRs in the framework of the DFT. In contrast to other manuscripts concerning the study of only one impurity substituted in the BNNRs, we have included the combined effect of the relative position of dopants and the sublattice where the C atoms are incorporated. In particular, we have found that substitutions on the boron lattice of the BNNRs and near the edge are the main energetic routes to stabilizing the C-doped BNNRs.

C-doping for either single and multiple boron/nitrogen substitutions induce spontaneous magnetization in the BNNRs, finding an important correlation between the nature of the species (B or N) where the dopant is located and their resulting electronic properties. On one hand, when B atoms replace C, it is observed that the $E_{F}$ shifts to higher energy, causing the occupation of states from the conduction bands. On the other hand, with substitutions on the N lattice, it is observed that the E_F_ shifts to lower energies with respect to the pristine one. The lowering of the $E_{F}$ in the C-doped BNNRs results from a positive charge doping (minus one electron per dopant atom). This behavior was observed for even and odd numbers of dopants, up to 6.5% doping concentration.

As already shown, C-doped BNNRs are expected to be electrophilic sites near the dopants, independently of their location along the ribbons. Overall, our calculations suggest that C-doped BNNRs may be employed to adsorb organosulfur molecules following a physisorption process. Particularly, the C-doped BNNRs with impurities substituted on the B lattice lead to complexes more energetically stable that those obtained on C-doped BNNRs with impurities substituted on the N lattice for the complexes formed between the sulfur atom of the organosulfur molecule interacting directly on the nanosurface. Our calculations suggest the following order in terms of the higher interaction energy involving the sulfur atom in the adsorbed state: DBT > 4,6-DMDBT > 4-MDBT > Thio > B-Thio. Since other geometries in the adsorbed state involve important interactions with the delocalized π systems of the organosulfur compounds, it is important to perform, as a second step, a molecular dynamics study in order to calculate the selectivity of the C-doped BNNRs towards the sulfur atom of the organosulfur compounds in a mixture hydrocarbon stream as well as the entropic, thermal and translational effects dependent of the temperature.

## Figures and Tables

**Table 1:** Cohesive per atom (Gibbs free) energy in eV of the pristine and C-doped BNNRs.

*M* × *N*	Pristine	BNNR_BE	BNNR_NE	BNNR_BC	BNNR_NC
12 × 2	6.32 (−5.55)	6.29 (−5.41)	6.30 (−5.39)	6.27 (−5.39)	6.29 (−5.38)
16 × 2	6.48 (−5.68)	6.45 (−5.57)	6.46 (−5.55)	6.44 (−5.56)	6.45 (−5.55)
24 × 2	6.65 (−5.82)	6.63 (−5.74)	6.63 (−5.73)	6.62 (−5.73)	6.63 (−5.73)
30 × 2	6.72 (−5.88)	6.71 (−5.82)	6.71 (−5.81)	6.70 (−5.81)	6.71 (−5.80)
16 × 4	6.53 (−5.74)	6.46 (−5.62)	6.47 (−5.61)	6.45 (−5.61)	6.46 (−5.61)

**Table 2:** <<1>> Interaction energy (in kcal/mol) of the most stable adsorption configuration when sulfur atom of the organosulfur compounds is perpendicularly oriented to the C-doped BNNRs, <<2>> Interaction energy (in kcal/mol) of the most stable adsorption configuration when hydrogen atoms of organosulfur compounds are perpendicularly oriented to the C-doped BNNRs.

Complex	Thio		B-Thio		DBT		4-MDBT			4,6-DMDBT	
	<<1>>	<<2>>	<<1>>	<<2>>	<<1>>	<<2>>		<<1>>	<<2>>	<<1>>	<<2>>
BNNR_BE	−16.6	−17.1	−11.8	−9.9	−13.1	−14.1		−15.6	−16.2	−18.4	−18.5
BNNR_BC	−15.1	−16.3	−13.3	−10.2	−18.6	−15.1		−16.4	−17.0	−18.2	−19.0
BNNR_NE	−12.6	−12.6	−6.9	−5.5	−18.0	−8.8		−11.0	−10.3	−13.6	−12.7
BNNR_NC	−12.1	−13.8	−12.3	−6.2	−23.2	−11.9		−15.2	−13.3	−14.9	−15.6

## Supplementary Materials

The following are available online at https://www.mdpi.com/2079-4991/9/3/452/s1, Figure S1: Spin density of the C-doped BNNRs: (**a**) BNNR_BEBE, (**b**) BNNR_BEBC, (**c**) BNNR_NENE (**d**) BNNR_NENC of size 12 × 2, (**e**) BNNR_4B and (**f**) BNNR_4N of size 30 × 2. Green, light blue, brown and pink spheres represent B, N, C and H respectively; Figure S2: Dual descriptor of the C-doped BNNRs (**a**) BNNR_BEBE, (**b**) BNNR_BEBC, (**c**) BNNR_NENE and (**d**) BNNR_NENC. Green, light blue, brown and pink spheres represent boron, nitrogen, carbon and hydrogen atoms respectively. The light blue lobes represent regions for nucleophilic attacks (*f*^2^(*r*) > 0) and the yellow lobes represent regions for electrophilic attacks (*f*^2^(*r*) < 0); Figure S3: (Color online): Complexes formed between B-Tio on the C-doped BNNRs (**a**) BNNR_NE, (**b**) BNNR_NC and the complexes formed between DBT on the C-doped BNNRs (**c**) BNNR_NE and (**d**) BNNR_NC respectively. Green, light blue, brown, yellow and pink spheres represent boron, nitrogen, carbon, sulfur and hydrogen atoms respectively.
