# Supplementary material for: Reactivity of Atomically Functionalized C-Doped Boron Nitride Nanoribbons and Their Interaction with Organosulfur Compounds
Source: Nanomaterials (Basel). 2019 Mar 18;9(3):452. doi: 10.3390/nano9030452 (PMC6474104; doi:10.3390/nano9030452)
Supplement: Supplementary file 1 [file nanomaterials-09-00452-s001.pdf]

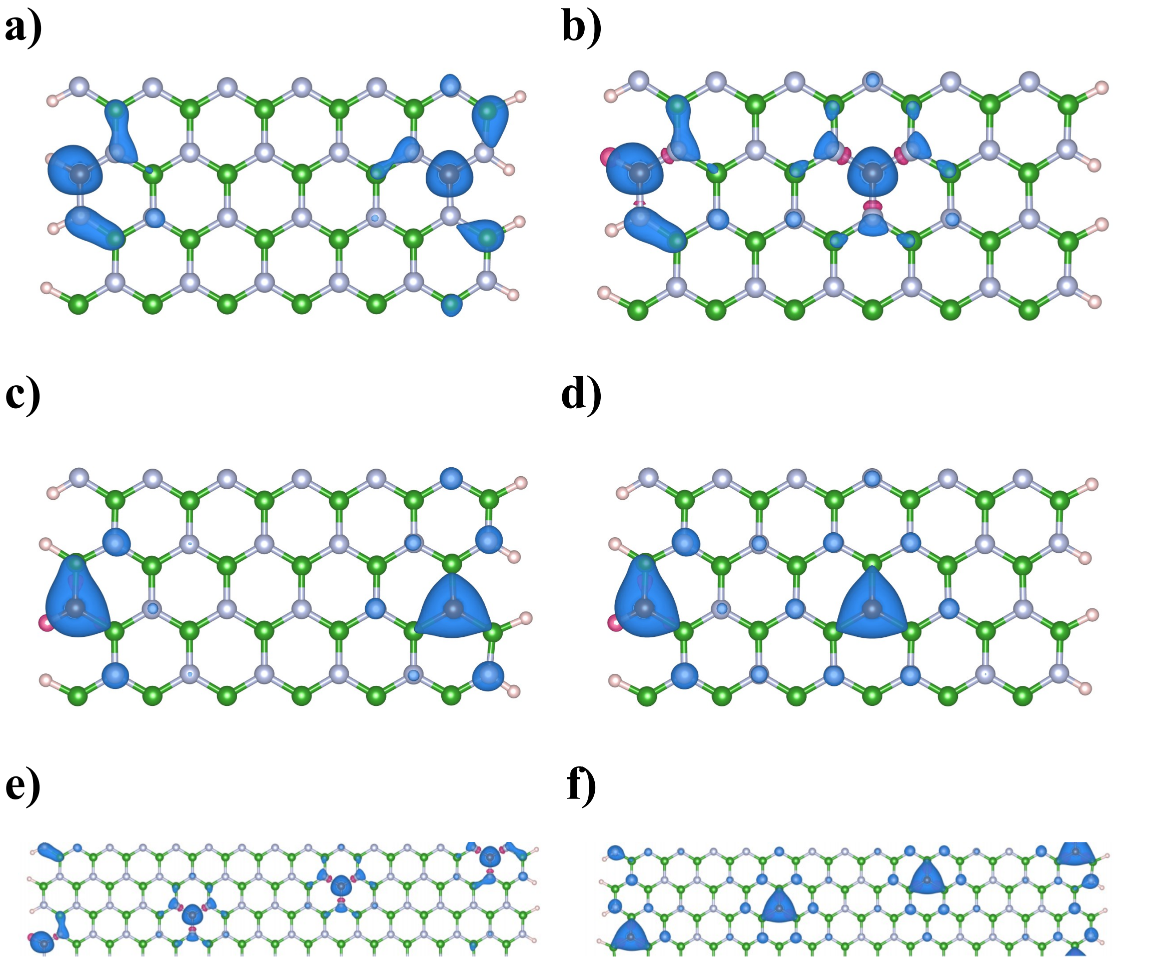

Figure S1 Spin density of the C-doped BNNRs: (a) BNNR_BEBE, (b) BNNR_BEBC, (c) BNNR_NENE (d) BNNR_NENC of size 12 × 2, (e) BNNR_4B and (f) BNNR_4N of size 30 × 2. Green, light blue, brown and pink spheres represent B, N, C and H respectively.

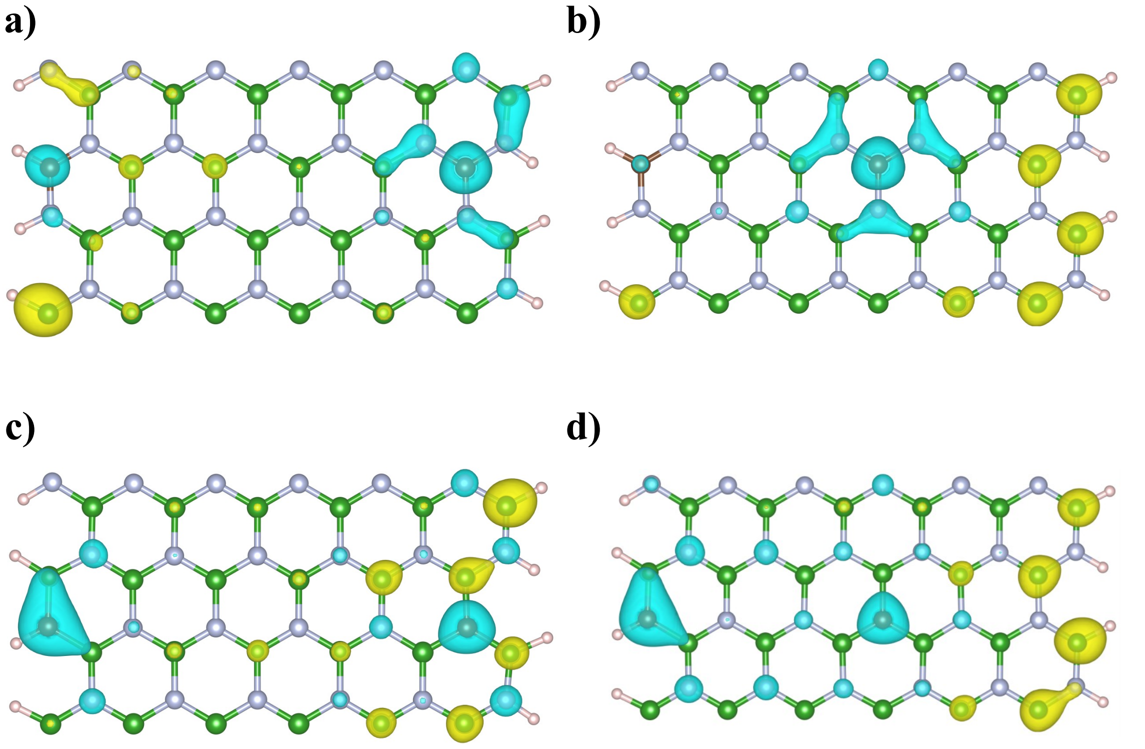

Figure S.2. Dual descriptor of the C-doped BNNRs (a) BNNR_BEBE, (b) BNNR_BEBC, (c) BNNR_NENE and (d) BNNR_NENC. Green, light blue, brown and pink spheres represent boron, nitrogen, carbon and hydrogen atoms respectively. The light blue lobes represent regions for nucleophilic attacks ($f^2(r) > 0$) and the yellow lobes represent regions for electrophilic attacks ($f^2(r) < 0$).

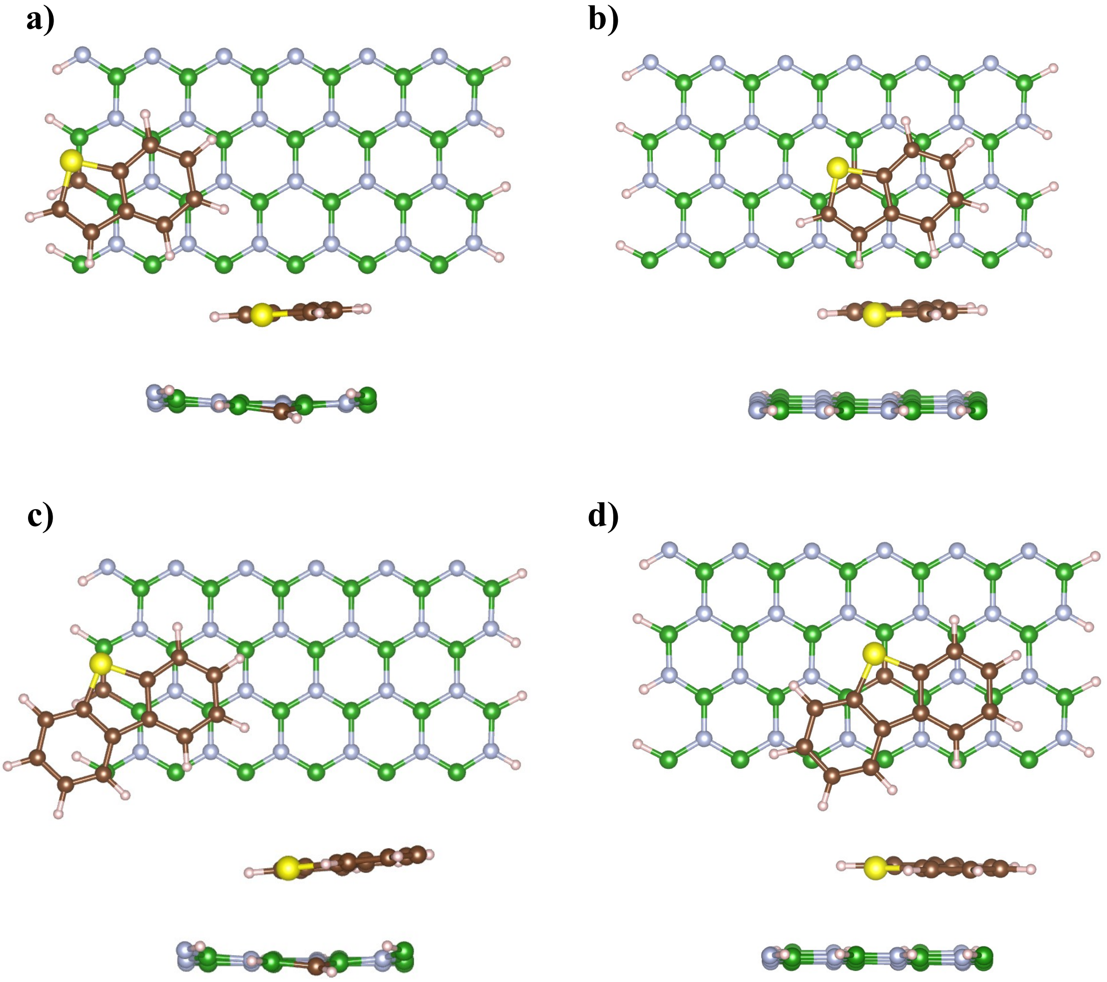

Figure S.3 (Color online): Complexes formed between B-Tio on the C-doped BNNRs (a) BNNR_NE, (b) BNNR_NC and the complexes formed between DBT on the C-doped BNNRs (c) BNNR_NE and (d) BNNR_NC respectively. Green, light blue, brown, yellow and pink spheres represent boron, nitrogen, carbon, sulfur and hydrogen atoms respectively.
